# A Potential Role of Esophageal Cancer Related Gene-4 for Atrial Fibrillation

**DOI:** 10.1038/s41598-017-02902-x

**Published:** 2017-06-02

**Authors:** Li Huang, Hua Yu, Xinrong Fan, Xue Li, Liang Mao, Jun Cheng, Xiaorong Zeng, Xitong Dang

**Affiliations:** Institute of Cardiovascular Research, the Key Laboratory of Medical Electrophysiology, Ministry of Education of China, Collaborative Innovation Center for Prevention and Treatment of Cardiovascular Disease of Sichuan Province, Southwest Medical University, Luzhou, Sichuan 646000 China

## Abstract

Epidemiological studies have shown a strong correlation between tumor and AF. However, the molecular link between tumor and AF remains unknown. ECRG4, a tumor suppressor gene that is expressed in the A-V node and in sporadic ventricular myocytes, inhibits tumorigenesis and monitors tissue homeostasis by functioning as a ‘sentinel’ molecule gauging inflammatory and cell proliferative responses. To explore the potential physiological function of Ecrg4 in heart, we evaluated its distribution in heart, analyzed its expression in patients with persistent AF and in a canine AF model, and dissected the molecular events downstream of Ecrg4. The results showed that the level of Ecrg4 expression is homogenously high in atria and the conduction systems and in sporadic ventricular myocytes. Importantly, the expression of Ecrg4 was significantly decreased in atrial appendages of AF patients than patients with SR. Moreover, in rapid pacing canine AF models, the expression of ECRG4 in atria was significantly decreased compared to that of the controls. Mechanistically, knockdown ECRG4 in atrial myocytes significantly shortened the APDs, inhibited the expression of Gja1, and activated pro-inflammatory cascades and genes involved in cardiac remodeling. These results suggest that Ecrg4 may play a critical role in the pathogenesis of AF.

## Introduction

Atrial fibrillation (AF), the most common form of cardiac arrhythmia, affects 1.5–2% population <65 years old and up to 10% in those >80 years old. It is becoming an epidemic as population ages^1^. Persistent or inadequately treated AF may cause blood coagulation in heart, leading to stroke, congestive heart failure, myocardial infarction and other grave medical conditions. AF is a heterogeneous disease and current treatment strategies aiming at symptom relief through rate and rhythm controls and prevention of complications are far from satisfactory and sometimes even precipitate arrhythmia occurrence^2^. Therefore, studies that explore the molecular mechanisms underlying AF are urgently needed.

Although significant progress has been achieved in the management of AF, the pathogenesis of AF remains incompletely understood. Traditional AF predisposing factors include ageing, cardiovascular diseases, lung disease, thyroid disease and cardiac surgery. Recently, some systemic diseases such as obesity, diabetes mellitus, tumor, inflammatory bowel diseases, gout, and even psoriasis have been associated with increased risk of AF. Most, if not all, these predisposing medical conditions have been found to have a sterile, persistent, and mild systemic inflammation^3^. Indeed, substantial evidence has shown that inflammation promotes the initiation and perpetuation of AF^4–7^. Atrial myocarditis was first discovered in patients with lone non-postoperative AF, which was not observed in subjects in Sinus rhythm^8^. Subsequently, epidemiological studies showed that serum C-reactive protein (CRP) and inflammatory cytokines such as TNFα, IL1, IL8, IL6, and monocyte chemoattractant protein-1 (MCP1) were significantly up-regulated in AF patients compared to those in Sinus rhythm. The increased level of CRP has been reported to predict the occurrence/re-occurrence of AF^9–11^. Consistent with the role of inflammation in the development of AF, drugs that have anti-inflammatory effect such as glucocorticoids, statins, poly-unsaturated fatty acid, colchicine, angiotensin converting enzyme inhibitors and angiotensin receptor inhibitors can reduce the risk of AF, and some have been used to prevent and even treat postoperative AF^12–15^. Furthermore, inflammatory mediators have been shown to disrupt calcium homeostasis, activate pro-fibrotic pathway, inhibit gap-junction intercellular communication (GJIC), and induce cardiomyocyte necrosis and apoptosis, which can all contribute to the formation of arrhythmogenic substrate^16–18^.

Recently, the association between the incidence of AF and cancer has attracted much attention^19^. In a case-control study, patients with AF had a 10-fold increased incidence of colorectal cancer compared to those without AF^20^. In a cohort study, patients with newly-diagnosed AF had a >5-fold increased cancer diagnosis than the expected incidence of cancer in general population in a 3-month follow-up^21^. The association was also supported by the finding that the incidence of AF in patients admitted for cancer surgery was >2 fold than those for non-neoplastic surgery^22^. These results warrant further investigation of the potential link between tumor and AF.

Esophageal Cancer Related Gene-4(Ecrg4) was originally characterized as a tumor suppressor gene^23^. It is constitutively expressed in many tissues at quiescent state and significantly decreased in cancer^24^. Ecrg4 encodes a pre-propeptide that is secreted but tethered on cell surface where it is colocalized with innate immunity complex TLR4-MD2-CD14^25^. Upon injury, Ecrg4 is shed immediately from the surface and its gene expression is rapidly decreased in 24 hours, which is accompanied by the activation of inflammatory cascades and tissue proliferative response^26^. Restoration of Ecrg4 significantly inhibited cell proliferation and migration, and decreased inflammation through suppression of the key inflammatory regulator, NF-κb and its downstream target gene, cyclooxygenase-2(COX2)^24, 26^. In addition, naïve T cells express high level of Ecrg4 that is down-regulated upon activation^27^. These discoveries suggest that constitutive expression of Ecrg4 in quiescent state monitors tissue homeostasis, whereas loss of Ecrg4 activates inflammatory and tissue proliferative responses that contribute to the development of tumor, wound resolution, and possibly atrial remodeling^28, 29^.

Ecrg4 is expressed in atrioventricular (A-V) node, atria, and sporadic ventricular myocytes^30, 31^. In light of the down-regulation of Ecrg4 and high incidence of AF in cancer patients, we hypothesize that Ecrg4 may play an important role in the pathogenesis of AF.

## Materials and Methods

### Materials

Immunohistochemistry (IHC) kit and 2 × PCR blue-mix were purchased from Shenggong Biotech Inc. (Shanghai, China). SYBR™ Green master mixes were from Thermo Fisher. Oligo nucleotides were synthesized by Qingke Biotech Inc. (Chengdu, China). Sodium pentobarbital was the product of Merck Inc. All other chemicals, unless specified otherwise, were from Sigma.

### Ethics

All animal studies were performed in accordance with the Chinese National Guidelines for the Use and Care of Experimental Animals. All protocols for animal studies were approved by the Experimental Animal Ethics Committee of Southwest Medical University. All methods for the human tissue harvest and use were performed in accordance with an approved protocol by the Ethics Committee of Southwest Medical University.

### Harvest of specimens of human atrial appendage

Human right atrial appendages were harvested from 5 patients with persistent AF and 5 age- and gender-matched patients in Sinus rhythm (SR) respectively from the affiliated hospital of Southwest Medical University, during heart surgery, and an informed consent was obtained prior to the surgery.

### Establishment of canine AF model

The AF model was established as described previously^32^. Briefly, adult mongrel dogs were anesthetized by injecting 30 mg/kg sodium pentobarbital intravenously, followed by implantation of a 6 F bipolar electrode in the right atria through the right femoral vein. AF group underwent rapid atria pacing at a frequency of 600 beats/minute for a total of 6 hours. Burst pacing (duration = 0.5 ms, interval = 100 ms, voltage = twice the diastolic threshold) was applied to atria before and 6 hour after the pacing to confirm the electrical atria re-modeling. Each burst lasted for 30 seconds, followed by a 2-minute interval. A total of 10 bursts were applied to each dog, and the EKGs were recorded using a multi-leads electrophysiological instrument (LEAD 7000A, Sichuan Jinjiang Electronic Science and Technology Co., Ltd. Chengdu). The criteria for a successful establishment of AF model were EKGs showing no P-wave, and f-wave of different sizes, intervals, and shapes that had lasted for at least 10 seconds. The duration of AF was defined as the time period from the successful induction of AF to the reversal of f-wave to the Sinus P-wave. A total of 7 AF models and 6 controls were used for gene expression analysis.

### Isolation of neonatal cardiomyocytes

Neonatal Spague-Dawley (SD) rats of 1–3 days old were operated to expose their hearts using thoracotomy. The hearts were harvested, and blood was rinsed with sterile PBS in a 10 cm cell culture dish. Atria and ventricles were identified under the magnifying microscopy and harvested separately. The specimens were flushed twice with PBS, transferred into a glass culture bottle, which were then digested by trypsin at 4 °C for 12–16 hours, followed by digestion with Collagenase II for 10 min at 37 °C. Single cells were collected and plated into 100 mm culture dishes or 6-well plates containing coverslips at appropriate density in 10% DMEM containing low glucose.

### Immunohistochemistry and immunofluorence

Nine SD rats of 200–250 grams were intramuscularly anesthetized with 3% pentobarbital sodium (30 mg/kg body weight) and decapitated, the hearts were quickly excised and placed into HEPES-buffered Tyrode’s solution (4 °C) consisting of the following components (in mM): NaCl 137.0, KCl 5.9, MgCl_2_ 1.2, CaCl_2_ 1.8, glucose 12.0, HEPES 10.0, titrated to pH 7.4 with NaOH. Atrium, ventricle, and Sinoatrial node (SAN) strip of each rat were dissected separately. The SAN strip, defined as the region bordered by its anatomic landmarks (the crista terminalis, the interatrial septum, and the superior and inferior vena cavae), was dissected from heart tissues. Tissues were washed twice with the Tyrode’s solution, and immediately fixed in 4% paraformaldeyhyde for overnight and embedded for tissue microtome. Tissues were sectioned at 4 mm and Immunohistochemistry was performed using the ICH kit (Shenggong Biotech Inc., Shanghai) following manufacturer’s instructions. The primary antibody was anti-Ecrg4 (Sigma) with a dilution of 1:500 and the secondary antibody was goat anti-rabbit-HRP at 1:10,000 dilution. Human atrial appendage specimens were processed the same way as that for adult rat heart. For immunofluorence of neonatal cardiomyocytes (CMs), newly-isolated CMs were seeded in 6-well plate containing coverslips and continued to incubate for overnight. The cells from 3–4 coverslips were then washed with PBS, fixed with 4% paraformadelhyde, permeabilized with 0.1% Triton X-100, and then proceeded for immunofluorence with anti-Ecrg4, followed by goat anti-rabbit Alexa Fluor 488 (Thermo Fisher), and nuclear counter-staining with DAPI (Thermo Fisher). The coverslips were then mounted and images were acquired with confocal microscopy.

### Construction of lentivirus expressing ECRG4 small RNA and knockdown ECRG4 in neonatal cardiomyocytes

Small RNAs targeting human ECRG4 were designed using the program (http://katahdin.cshl.org:9331/homepage/siRNA/RNAi.cgi?type=shRNA) recommended in the pGreenPuro^TM^ manual (System Biosciences). The two oligos (designated as E16 and E25) containing the core siRNA sequences, 5′-TGGCCGTTGATGAGAATAAAG-3′ and 5′-ACTACCAACGTCACTATGATG-3′ targeting ECRG4 coding sequence, were annealed with their corresponding reverse complementary strands and cloned into pGreenPuro, a bicistronic lentiviral vector co-transcribing GFP downstream of the siRNA precursor. The identity of each plasmid was confirmed by DNA sequencing. The knockdown efficiency was determined by transient transfection of PC3 cells, a prostate cancer cell line, and the expression of Ecrg4 was evaluated by real-time PCR. E16 had a better knockdown efficiency and was used in the knockdown experiment. A plasmid containing siRNA targeting firefly luciferase (Luc-siRNA) was used as a control. Lentiviruses were packaged using pPACKH1 Lentivector Packaging Kit (SBI, Cat #LV500A-1) and tittered by flow cytometry using limiting dilution. About 3 × 10^5^ PFU virus was used to transduce neonatal CMs in the presence of 4–8 μg/ml Polybrene in a 6-well plate containing coverslips for 36 hours. The coverslips were used for patch-clamping to record action potential (AP) and the cells on the edge of the wells were used to confirm the knockdown of ECRG4 and to evaluate the expression of genes commonly involved in inflammation and atria remodeling by real-time PCR.

### Action potential recording using patch-clamp

Action potential was recorded using patch-clamp amplifier (EPC-10, HEKA, and Germany). Single-pipette whole-cell patch-clamp technique under current clamp configuration was used to record the APs of single cardiomyocyte. The pipette solution contained (in mmol/L): KCl 140, MgCl_2_ 1, HEPES 5, Glucose 10, EGTA 10, Mg_2_ATP 5, GTP 0.1, phosphocreatine 5 (pH 7.2, adjusted with KOH). The bath solution contained (in mmol/L): NaCl 137, KCl 5.4, MgCl_2_ 1.2, NaH_2_PO_4_ 1.2, HEPES 20, Glucose 10, CaCl_2_ 1.5 (pH 7.4, adjusted with NaOH). APs were elicited by depolarizing pulses with width of 2 ms, intensity of 800 pA and frequency of 1 Hz. Data were sampled at 20 kHz and filtered at 1 kHz. A total of 8 GFP-positive CMs from Ecrg4-siRNA and Luc-siRNA knocking down, respectively, were used to record APs. Action potential durations at 50% repolarization (APD_50_) and 90% repolarization (APD_90_) were analyzed. All the experiments were conducted at room temperature (22–25 °C).

### Gene expression analysis

After harvesting, rat and canine hearts were washed with Tyrode’s solution, and atria, ventricles, and Sinoatrial node (SAN) strips were dissected separately, which were immediately snap-frozen in liquid nitrogen and stored at −80 °C freezer. For RNA preparation, neonatal CMs were immediately processed after experiments and frozen tissues were first grinded with an electric grinder, followed by total RNA extraction with Trizol following the protocol recommended by the vendor (Life Technologies). One μg of total RNA was reverse transcribed (BioRad) in 20 μl reaction containing 5 μl of 5x reaction buffer and 1 μl of reverse transcriptase. Of the 20 μl cDNA, 1 μl was used for real-time PCR. Real-time PCR was performed in a 25 μl of reaction consisting of 12.5 μl CYBR mix, 1 μl of each primer at 10 μM, 1 μl of cDNA, and 9.5 μl of water. The cycler conditions for both genes of interest and GAPDH were 95 °C for 5 min., 45 cycles of 94 °C for 30 sec., 62 °C for 30 sec., and 72 °C for 30 sec. The PCR efficiency for all primer pairs were greater than 95%. Relative expression was calculated using ΔΔCt method as described in BioRad’s real time PCR manual. Primers for ECRG4 are sense: 5′-CCCAGGTGGCATAAGTGGAA-3′ and anti-sense: 5′-ACTGCTGGTACCACTGCTGCACC-3′. Primers for GAPDH are sense: 5′-GAGCGAGATCCCTCCAA-3′ and anti-sense: 5′-ACTGTGGTCATGAGTCCTTC-3′. Primers for others are available upon request.

### Statistical analysis

The data, expressed as ‘Mean ± SD’, were analyzed using Student’s t test with SPSS19.0 software. *P < 0.05 was considered to be statistically significant.

### Data availability statement

Data published in this article are available upon request.

## Results

### Distribution of Ecrg4 in heart

**Figure 1:**
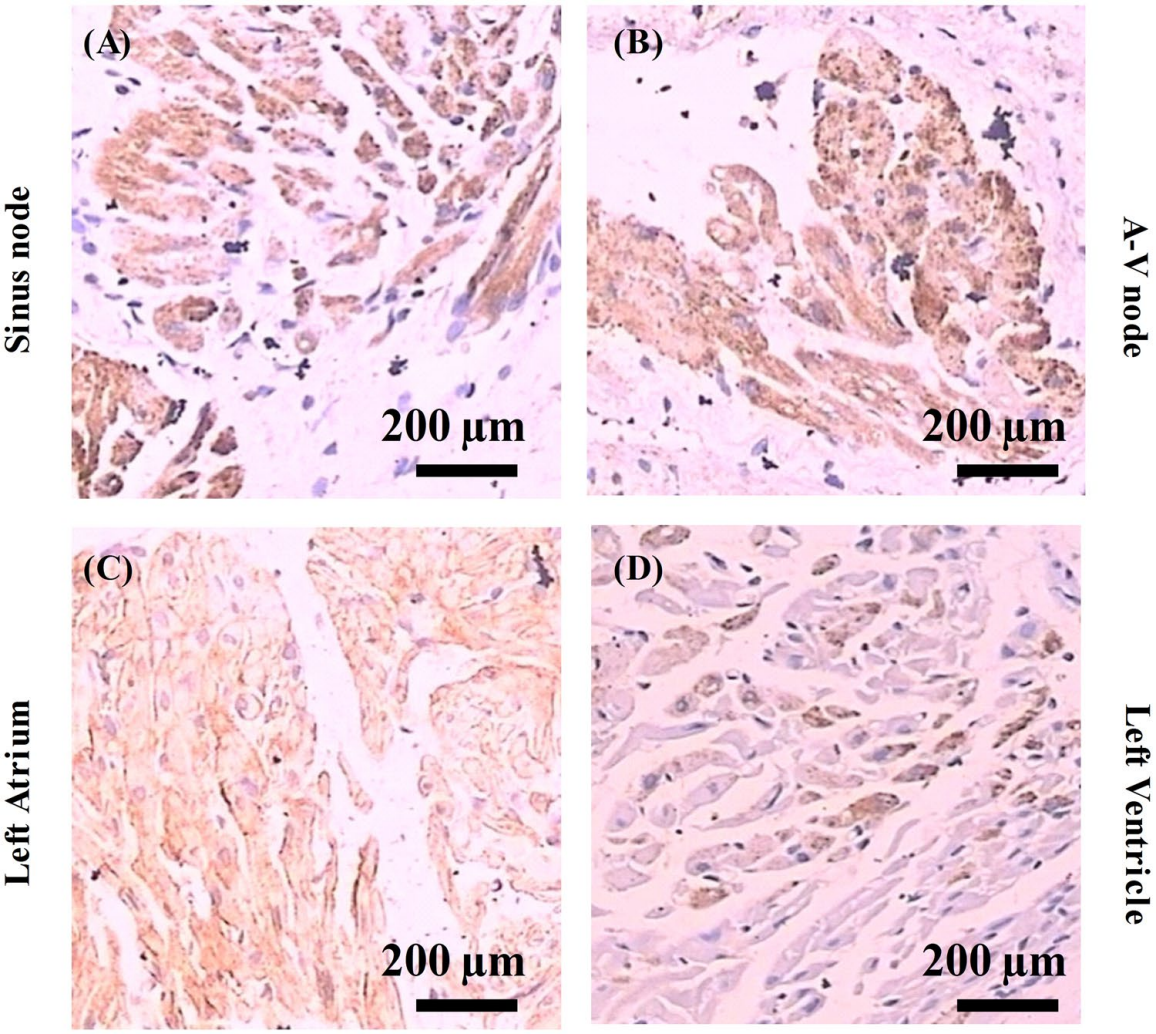
Distribution of Ecrg4 in adult rat heart by immunohistochemistry. Specimens of Sinus node, A-V node, left atrium, and left ventricle were dissected from adult SD rat hearts, and processed for IHC as described. Representative images showing that Ecrg4 (brown staining) is expressed in Sinus node (**A**), A-V node (**B**), left atrium (**C**), and sporadically in ventricular myocytes of left ventricle (**D**). Scale bar, 200 µm.

**Figure 2:**
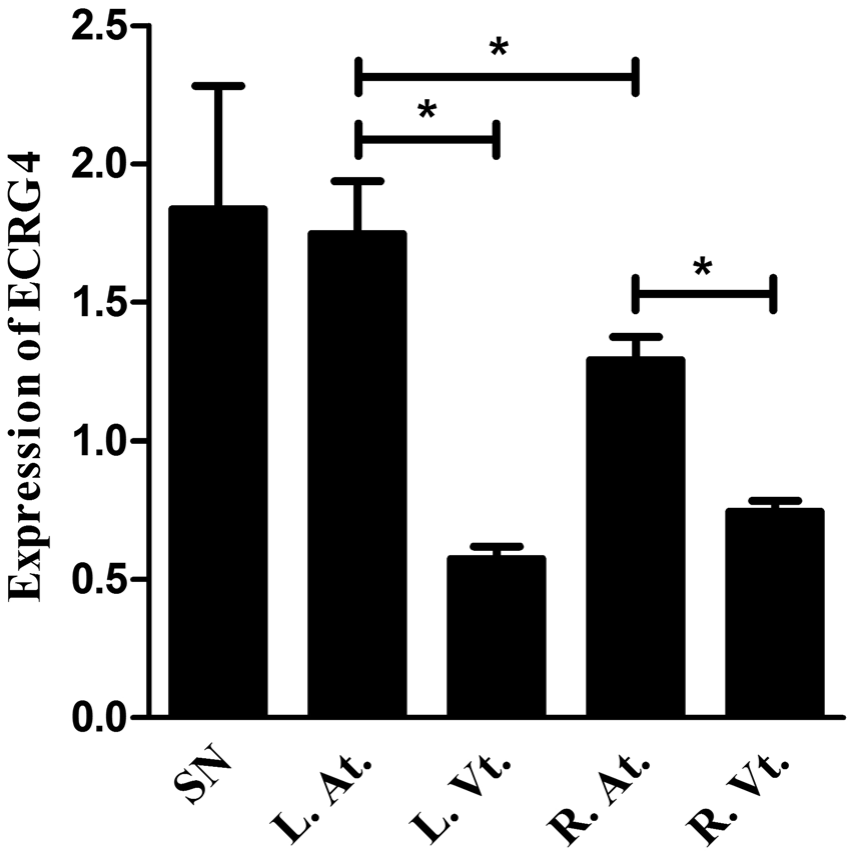
Distribution of Ecrg4 in adult rat heart by real-time PCR. Hearts were harvested from 9 adult rats, specimens of Sinus node, left and right atria (L. and R. At.), and left and right ventricles (L. and R. Vt.) were dissected, which were processed for total RNA isolation and real-time PCR as described in materials and methods. SN and L. At. express the highest level of ECRG4, followed by R. At. and both ventricles. Atria express significantly higher level of ECRG4 than ventricles (n = 9, P < 0.05), and L. At. express higher level of ECRG4 than R. At. (n = 9, P < 0.05). Data were presented as “Mean ± SD”, and the experiments were performed in triplicate and repeated three times.

It has been reported that Ecrg4 is expressed in the A-V node and sporadic ventricular myocytes of adult rat heart^30, 31^. To explore the potential function of Ecrg4 in heart, we analyzed its distribution in adult SD rat heart by immunohistochemistry (IHC) and real-time PCR. Rats were anesthetized and hearts were harvested, washed in PBS, and dissected into Sinus node, left and right atria, and left and right ventricles. Each tissue was split into two parts, one for IHC and the other for real-time PCR. Figure 1 are representative IHC images showing a homogenous stronger Ecrg4 immunopositive staining (brown color) in Sinus node (1A), A-V node (1B) and left atrium (1C), and sporadic strong Ecrg4 immunopositive myocytes (1D) can be seen in left ventricle. When the expression of ECRG4 was evaluated by real-time PCR (n = 9) (Fig. 2), Sinus node (SN) and left atria (L. At.) express the highest level of ECRG4, followed by right atria (R. At.), and then right and left ventricles (R. Vt. and L. Vt.). Atria express significantly higher level of ECRG4 than that of ventricles (P < 0.05), and left atria express higher level of ECRG4 than that of right atria (P < 0.05).

### Neonatal cardiomyocytes express higher level of Ecrg4 in atria than ventricles

**Figure 3:**
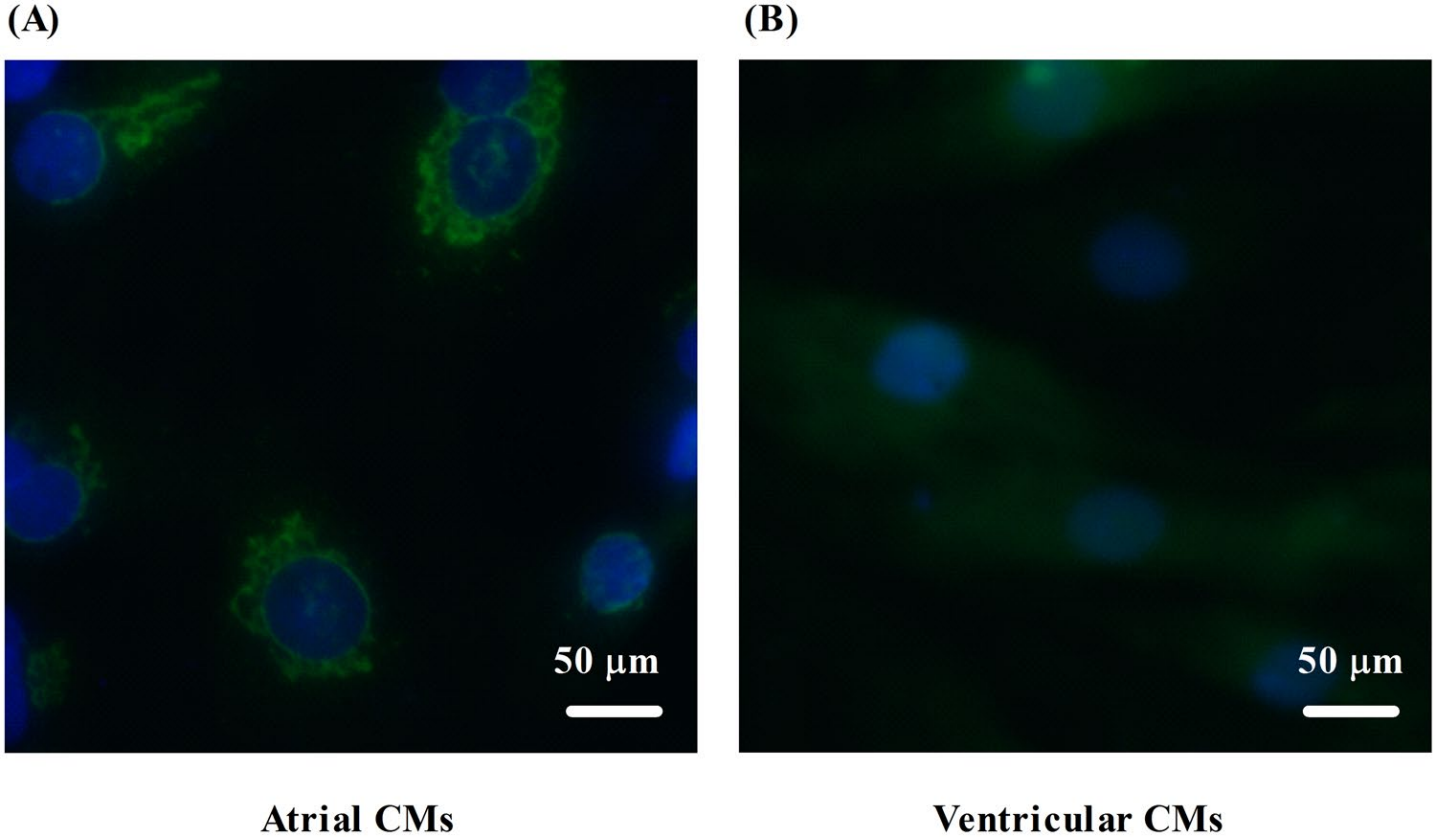
Ecrg4 is expressed in neonatal rat cardiomyocytes. Neonatal atrial and ventricular myocytes were prepared separately as described in materials and methods, which were seeded in a 6-well plate containing coverslips for overnight. Cells were fixed, permeabilized, and proceeded for immunofluorence using anti-Ecrg4 as primary antibody, goat anti-rabbit Alexa Fluor 488 as secondary antibody, and DAPI for nuclear staining. Representative images showing relative higher level of Ecrg4 (bright green) in perinuclear region and in endoplasmic reticulum in neonatal atrial myocytes (**A**, left panel) compared to relative lower level of Ecrg4 (faint green) diffused in the cytoplasm of ventricular myocytes (**B**, right panel). Scale bar = 50 µm.

To confirm that atria express relatively higher level of Ecrg4 than that of ventricles, neonatal rat atrial and ventricular myocytes were isolated separately and expression of Ecrg4 was evaluated by immunofluorence. As shown in Fig. 3, a much stronger Ecrg4-positive immunofluorence signal (green color) localized to peri-nucleus and endoplasmic reticulum (ER) regions was seen in atrial myocytes (A, left panel) compared to a faint homogeneous immunofluorence in the cytoplasm of ventricular myocytes (B, right panel). This result suggests that atria express higher level of Ecrg4.

### Expression of Ecrg4 was down-regulated in appendages of AF patients

**Figure 4:**
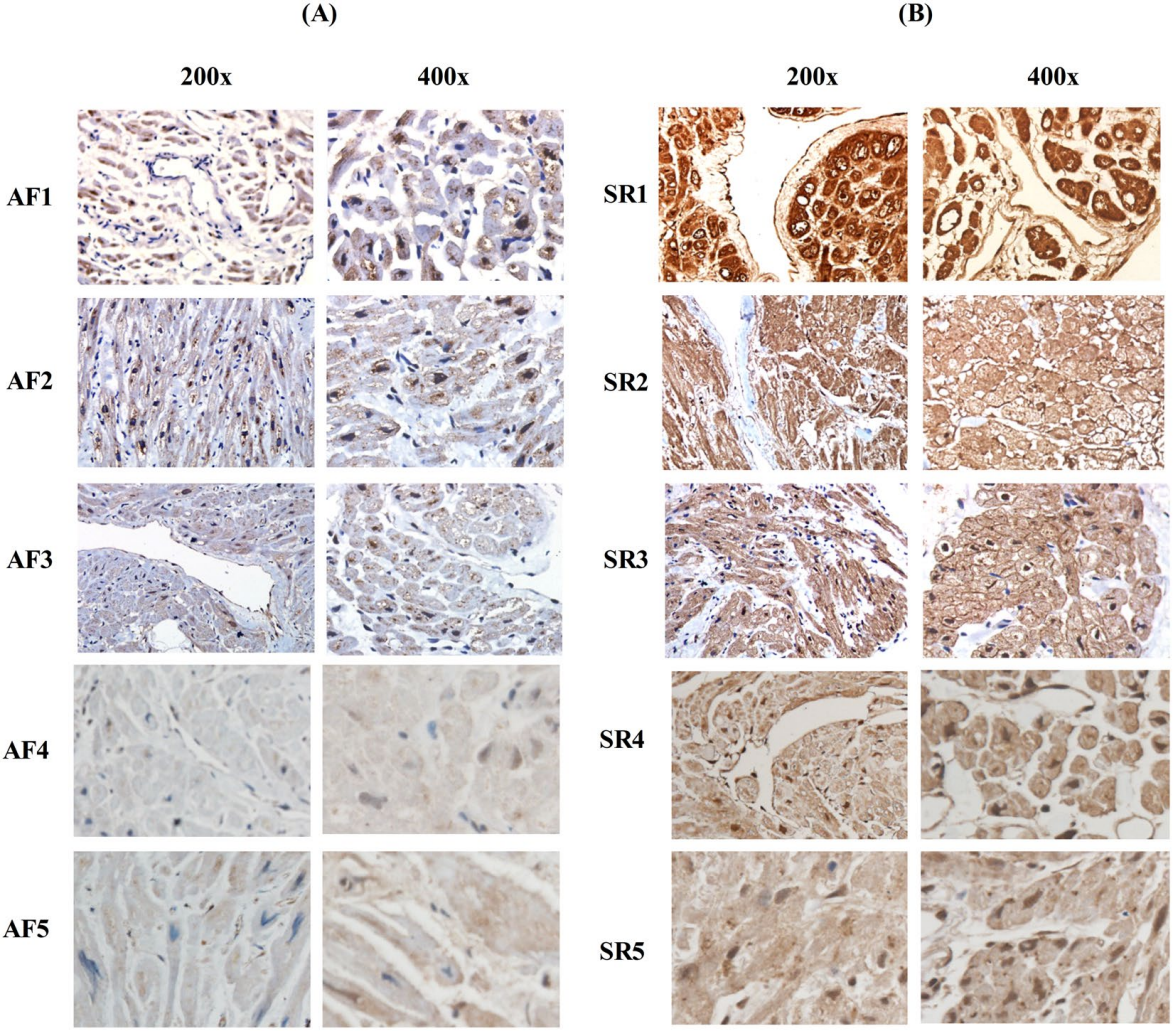
Expression of Ecrg4 is down-regulated in appendages of AF patients. Specimens of atrial appendage from right atrium were harvested from 5 RHD patients with persistent AF (**A**, labeled AF1–5 on left panel) and 5 RHD patients with SR (**B**, labeled SR1–5 on right panel) respectively. The specimen were fixed, microtomed, and proceeded for IHC using anti-Ecrg4 as primary antibody and goat anti-rabbit-HRP as secondary antibody per instructions in materials and methods. Representative images (with 200x magnification on the left side and 400x magnification on the right side of **A** and **B** respectively) showing a light brown staining of cardiomyocytes (low level of Ecrg4) in all 5 specimens of atrial appendages from patients with AF (**A**, left panel) relative to a dark brown staining of cardiomyocytes (high level of Ecrg4) in all 5 specimens of atrial appendages from patients with SR (**B**, right panel).

To explore the clinical relevance of Ecrg4 expression in atria and the conduction systems, we analyzed the expression of Ecrg4 by IHC in patients of rheumatic heart disease (RHD) with or without AF. Specimens of atrial appendage of 5 female patients with persistent AF (labeled AF1, 2, 3, 4, and 5) and 5 age-, and gender-matched patients with Sinus rhythm (SR, labeled SR1, 2, 3, 4, and 5) were harvested after surgery. Table 1 lists the age and the diagnosis of each patient. Specimens were fixed, paraffin embedded, microtomed, and proceeded for IHC as described in materials and methods. As shown in Fig. 4, the Ecrg4-specific immunoreactive staining shown as brown color, with 200x magnification on the left side and 400x magnification on the right side of (A) and (B) respectively, was significantly less intense in the appendages of patients with persistent AF (A, left panel) than control patients with SR (B, right panel), suggesting that Ecrg4 may participate in the pathogenesis of AF.

**Table 1:** Characteristics of Patients.

Patients	Age (years)	Comorbidities
AF1	43	RHD, MS
AF2	50	RHD, MS
AF3	42	RHD, MS
AF4	47	RHD, MS, MR
AF5	51	RHD, MS, AS, MR, TR
SR1	48	RHD, VD, AS, MR
SR2	40	RHD, CAD, MR
SR3	45	RHD, CAD, MS
SR4	50	RHD, VD, AS, AR, MR, TR
SR5	46	RHD, MVP, MR, TR

Abbreviations: RHD, rheumetic heart disease, MS, mitral valve stenosis, VD, valvular disease, AS, aortic stenosis, AR, aortic regurgitation, MR, mitral valve stenosis, CAD, coronary artery disease, MR, mitral valve regurgitation, TR, Tricuspid regurgitation, MVP, Mitral valve prolapse.

### Expression of Ecrg4 was down-regulated in atria in a canine AF model

**Figure 5:**
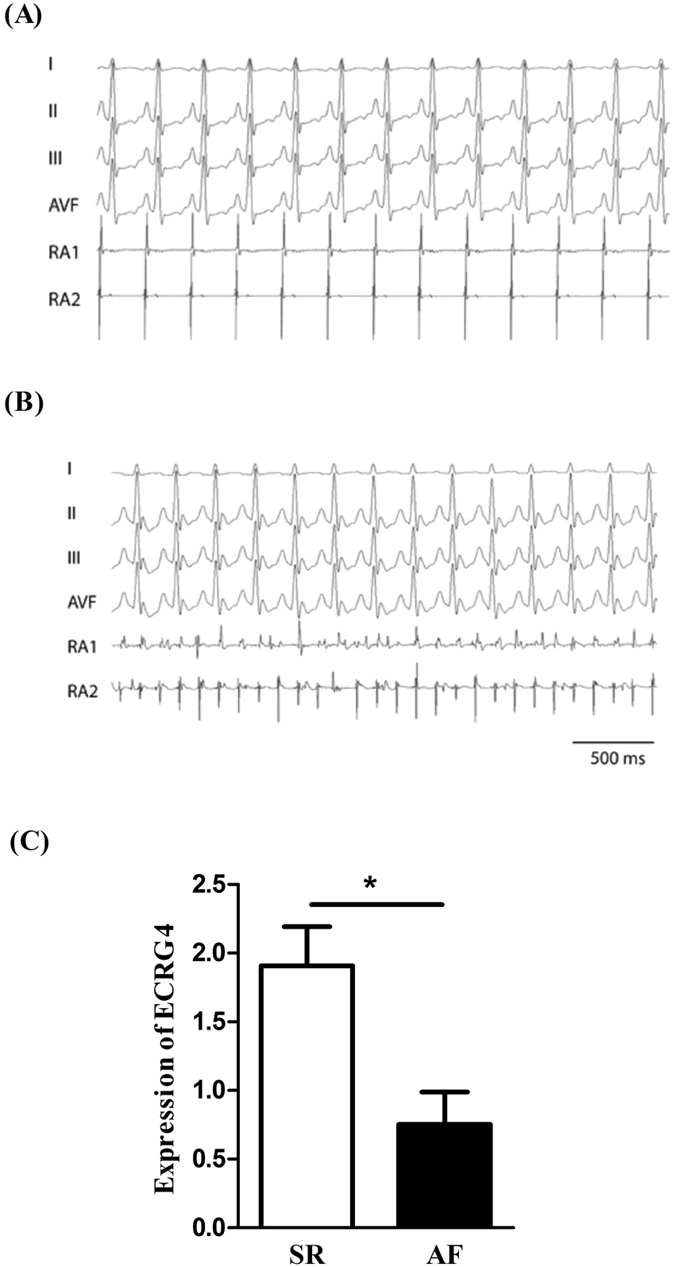
Establishment of canine AF model and expression of Ecrg4 in atria. AF was induced in canines using rapid atria pacing as described in materials and methods. AF was confirmed using surface and intracardiac electrograms showing a normal regular P and atrial waves (**A**) in a control dog relative to fast and irregular atrial waves (**B**) in an AF model dog. When gene expression was analyzed by real-time PCR (**C**), ECRG4 was significantly down-regulated (n = 8, P < 0.05) in AF models (AF, black bar) compared to controls in SR (SR, Open Bar). Data were presented as “Mean ± SD”, and the experiments were performed in triplicate and repeated three times.

To confirm the potential role of Ecrg4 in the pathogenesis of AF, a canine AF model was established by rapid atria pacing (RAP) as described previously^32^ and the successful establishment of AF model was confirmed by burst pacing as described in materials and methods. After continuous RAP for 6 hours, dogs were euthanized and left atrial appendages were collected for gene expression analysis. Figure 5A shows representative recordings of the regular normal AP waves in a control dog and 5B shows fast and irregular atrial waves in an AF model induced by a burst pacing. Wthen the expression of ECRG4 was analyzed by real-time PCR (Fig. 5C), it showed that ECRG4 was significantly down-regulated in AF models (black bar, n = 8) compared to control dogs (Open Bar) (P < 0.05). These results indicate that Ecrg4 may participate in the development of AF.

### Knockdown Ecrg4 affects the APDs and genes commonly involved in atrial remodeling

**Figure 6:**
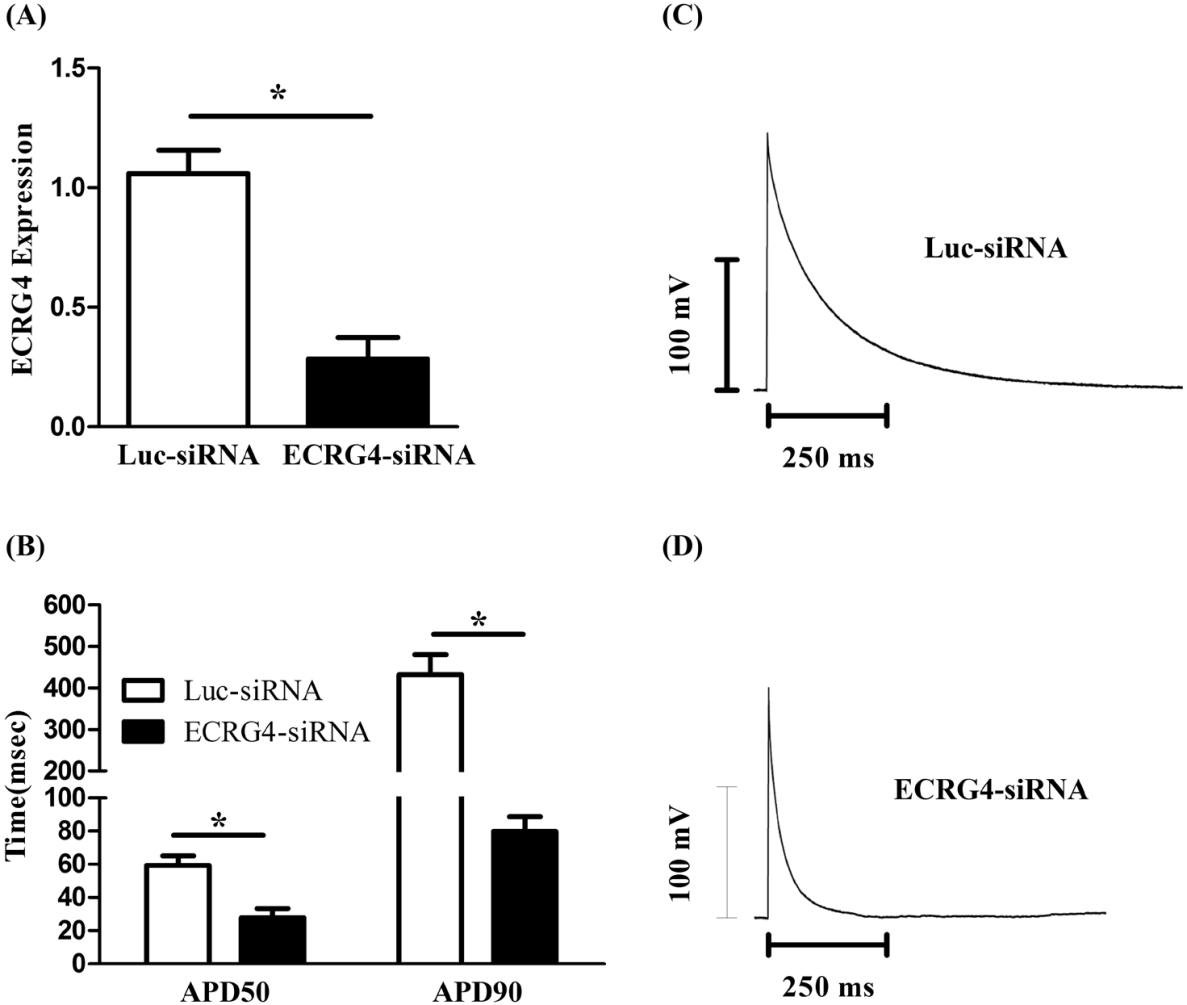
Knockdown ECRG4 in neonatal atrial myocytes shortens APD. Neonatal atrial CMs were transduced with lentivirus expressing Ecrg4-siRNA or luciferase-siRNA for 36 hours and the knock down of ECRG4 was confirmed by real-time PCR showing a 74% decreased ECRG4 expression in Ecrg4-siRNA (black bar) relative to Luc-siRNA transduction (open bar) (**A**, P < 0.05). Thirty-six hours after transduction, AP was recorded using patch-clamp on GFP positive CMs. Representative recordings showing APs in Luc-siRNA (**C**) and Ecrg4-siRNA (**D**) transduced CMs. Quantitative analysis of APD_50_ (**B**, left panel) and APD_90_ (**B**, right panel) showed 53% and 81% decrease, respectively, in Ecrg4-siRNA (black bars) relative to Luc-siRNA (open bar) transduced atrial myocytes (n = 8, P < 0.05 in both APD_50_ and APD_90_). Data were presented as “Mean ± SD”, and the knockdown experiment was performed in triplicate and repeated three times.

**Figure 7:**
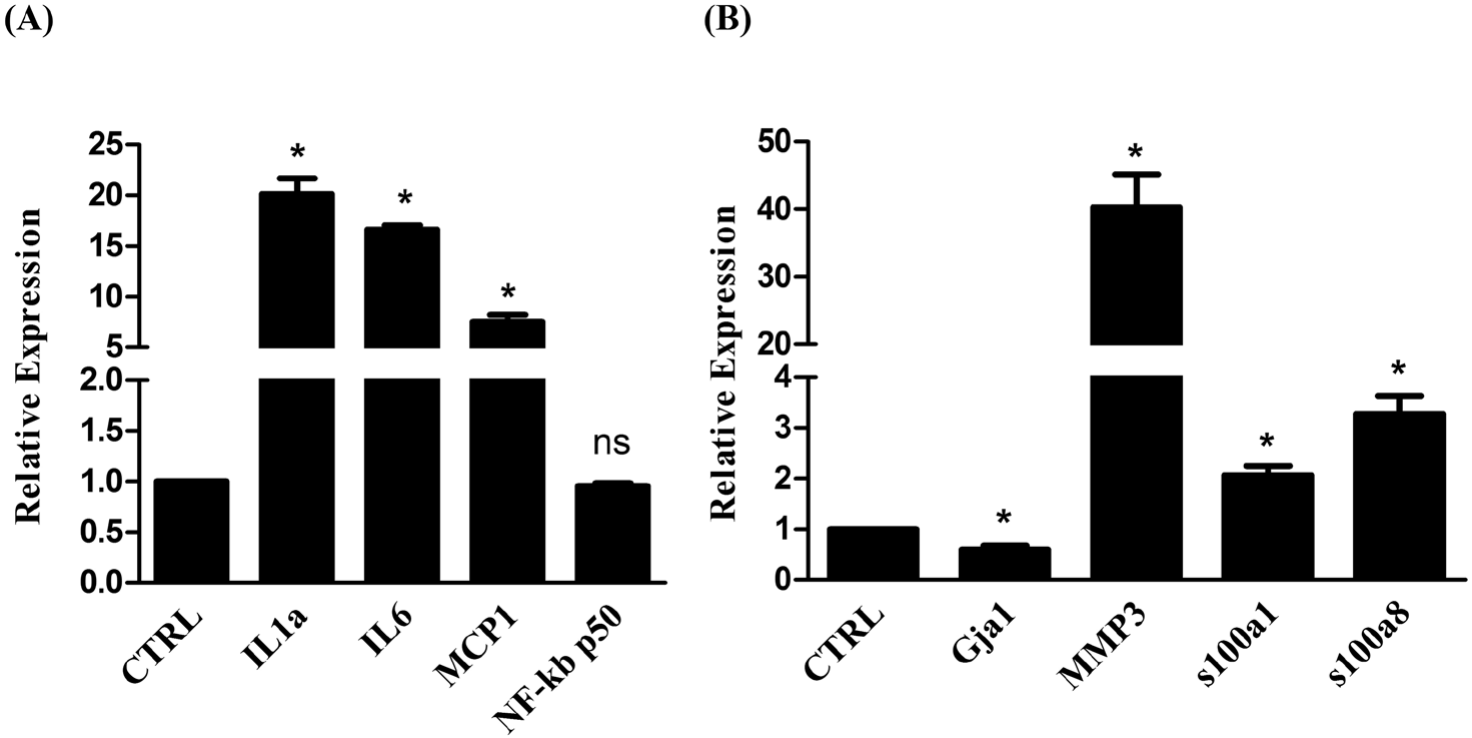
Knockdown ECRG4 affects the expression of genes implicated in inflammation and cardiac remodeling. Knockdown ECRG4 was performed as described in materials and methods and gene expression normalized to GAPDH was expressed as relative expression of the knockdown using Luc-siRNA, which was arbitrarily set as 1. Knockdown ECRG4 significantly increased the expression of IL1a, IL6, and MCP1 (**A**, P < 0.05 in all) but not that of NF-kb p50 (ns, non-significant) (**A**), inhibited the expression of Gja1 (**B**, P < 0.05) and activated the expression of MMP3, s100a1 and s100a8 (**B**, P < 0.05 in all). Data were presented as “Mean ± SD”, and the experiments were performed in triplicate and repeated three times.

To explore the potential molecular mechanisms of Ecrg4 in the pathogenesis of AF, neonatal atrial myocytes were seeded in a 6-well plate containing coverslips, and transduced on the next day with lentiviruses expressing Ecrg4-siRNA to knockdown Ecrg4 or luciferase-siRNA (Luc-siRNA) as a control as described in materials and methods. Cells on the coverslips were used for action potential recording, and cells on the edge of each well were used for real-time PCR confirming the knockdown of ECRG4 and analyzing the expression of pro-inflammatory cytokines and genes commonly involved in atrial remodeling. Thirty six hours after lentivirus transduction, the expression of ECRG4 was 74% lower in Ecrg4-siRNA (A, black bar) than Luc-siRNA (A, open bar) transduced atrial myocytes (P < 0.05). Figure 6C is a representative AP recording curve from Luc-siRNA knockdown and Fig. 6D is a representative AP recording curve from Ecrg4-siRNA knockdown atrial myocytes. When AP durations were analyzed, there were a 53% and 81% decrease of APD_50_ (Fig. 6B, left panel) and APD_90_ (Fig. 6B, right panel) respectively in Ecrg4-siRNA (black bars) compared to Luc-siRNA (open bar) transduced atrial myocytes (P < 0.05 in both APD_50_ and APD_90_). To further identify the downstream targets of Ecrg4 in AF, we profiled genes commonly implicated in inflammation and in cardiac remodeling. Compared to Luc-siRNA (arbitrarily set as 1), knockdown ECRG4 significantly up-regulated the expression of proinflammatory genes IL1a, IL6, MCP1 (P < 0.05 in all) but not that of NF-kb p50 (ns, non-significant) (Fig. 7A), down-regulated Gap junction alpha-1 protein (Gja1) (P < 0.05), and up-regulated MMP3, s100a1 and s100a8 (Fig. 7B) (P < 0.05 in all). These results suggest that Ecrg4 may play a critical role in the pathogenesis of AF, and loss of Ecrg4 may initiate the cascades of atrial remodeling.

## Discussion

The findings presented here show that Ecrg4 is expressed in Sinus node, A-V node, atrium and sporadic CMs of ventricle (Fig. 1), and levels of ECRG4 expression are higher in SN and atria than ventricles (Fig. 2A). In neonatal CMs, expression of Ecrg4 in atrial myocytes seems high and localized in the endoplasmic reticulum versus low and homogenous cytoplasmic distribution in ventricular myocytes (Fig. 3). Significantly, expression of Ecrg4 in atrial appendages was significantly lower in patients with persistent AF than patients with SR (Fig. 4). Moreover, in a canine AF model induced by rapid atria pacing, expression of ECRG4 in atria was significantly decreased compared to that of controls (Fig. 5C). Finally, knockdown ECRG4 in neonatal atrial myocytes significantly shortened the APD_50_ and APD_90_ (Fig. 6B–D), increased the expression of pro-inflammatory cytokines IL1a, IL6, and MCP1 (Fig. 7A), decreased the expression of Gja1, and increased the expression of MMP3, s100a1 and s100a8 (Fig. 7B). These discoveries point to the involvement of Ecrg4 in the pathogenesis of AF.

Since the cloning of ECRG4 from epithelial cells of normal esophagus^23^, Ecrg4 has now been characterized as a tumor suppressor gene that is constitutively expressed in normal tissues of many organs^24, 26, 29, 33^. Ecrg4 is not only expressed in the typical epithelial cells, but also expressed in specialized epithelial-derived cells including pituicytes, oligodendrocytes, choroid plexus epithelium, middle ear mucosa, cornea and airway epithelia, and even in adrenal gland and human leukocytes^34^. These wide tissue distributions are in consistent with the multiple tissue-dependent functions of Ecrg4. Mirabeau first reported that Ecrg4 was expressed in A-V node of heart by *in situ* hybridization^31^. Later, Porzionato *et al*. showed that Ecrg4 is homogenously expressed in atria and in sporadic ventricular myocytes of rat by immunohistochemistry^30^. Using both immunohistochemistry and real-time PCR, we showed that expression of Ecrg4 is high in Sinus node, A-V node and atria relative to ventricles, which agrees with microarray data that ECRG4 expression was higher in artial chamber/atria than ventricles during mouse embryo development (PubMed GEO Profile ID: 4938867) and in adult rats (PubMed GEO Profile ID: 62698595). Together, these discoveries suggest that Ecrg4 may play a critical role in normal cardiac physiology.

Epidemiogical studies have demonstrated a strong correlation between tumor and the incidence of AF. Predisposing factors for cancer patients to develop AF include advanced age, chronic inflammation, and cancer-induced dys-regulation of metabolism, radio- and chemo-therapy, disturbance of autonomous nerve system caused by pain and psychological stress, and surgery. However, whether tumor suppressor genes play a role in the pathogenesis of AF remains largely unknown. Kim *et al*. compared gene expression profiles in human atrial tissues between patients with chronic AF and those with Sinus rhythm (SR) and reported that tumor suppressor, p27 expression was significantly lower in patient with AF than patients with SR^35^. Genome-wide association studies (GWAS) have also associated transcription factor, zinc finger homeobox 3 (ZFHX3) with AF^36, 37^. ZFHX3 is a well-known tumor suppressor gene encoding AT-motif binding factor-1 (ATBF1)^38^. Rapid electric stimulation (RES) significantly activated STAT3 signaling pathway that has been shown to contribute to atrial remodeling^39^. Knockdown ZFHX3 in atrial myocytes activated STAT3, whereas over-expression of ZFHX3 inhibited the activation of STAT3 induced by RES, supporting the involvement of ATBF1 in the pathogenesis of AF^40, 41^. Moreover, ATBF1 interacts with Runt domain transcription factor 3 (Runt-3), and upon activation of TGFβ signaling pathway the ATBF1-Runt3 complex translocates to nucleus, where it transactivates pro-fibrotic genes, leading to increased fibrosis^42^. We showed that expression of Ecrg4 is decreased in atrial appendages of AF patients and in atria of a canine AF model, and knockdown ECRG4 in atrial myocytes significantly increased the expression of pro-inflammatory cykotines (IL1a, IL6, MCP1), down-regulated Gap junction alpha-1 expression (Gja1), and up-regulated the expression of s100a1, s100a8, and MMP3. S100a1 and s100a8 are members of the s100 calcium binding protein family expressed in cardiac muscle cells, which are critical regulators of cardiomyocyte Ca^2+^ cycling^43^. Together, these results support that the constitutively expressed Ecrg4 in atria monitors atrial homeostasis, and loss of Ecrg4 may promote the formation of arrhythmogenic substrate, increasing the risk of AF.

Rapid electric stimulation of cultured atrial myocytes produces electrical remodeling that mimic the typical features of atrial tachycardia remodeling *in vivo*. Yang *et al*. demonstrated that rapid electric stimulation of HL1 cells for 24 hours significantly decreased ECRG4 expression^44^, suggesting the involvement of Ecrg4 in atrial electric remodeling. In agreement, we found that knockdown ECRG4 in atrial myocytes significantly shortened APD. Atrial electric properties are maintained by the tightly-regulated calcium level and the coordination among ion channels. The shortening of APD can be caused by either increased outward current carried mainly by K^+^ and/or decreased inward current carried by Ca^2+^ during repolarization^45^. The decrease of APD can facilitate initiation and maintenance of reentrant circuits in AF^46, 47^. Knockdown ZFHX3 in atrial myocytes significantly abbreviated the APD, which were attributed to the increased expression of sarco/endoplasmic reticulum Ca^2+^-ATPase 2a (SERCA2a), ryanodine receptor, Kv1.4, Kv1.5, and Kir3, leading to larger SERCA2a activity, ultra-rapid delayed rectifier potassium currents, and transient outward currents^46^. However, the molecular mechanisms underlying the shortened APD in ECRG4 knockdown remain to be studied.

In summary, we have provided evidence to show that Ecrg4 is constitutively expressed in atria and the conduction systems and is down-regulated in AF. Moreover, loss of Ecrg4 modulated the expression of molecules promoting the formation of arrhythmogenic substrate, which tends to support that Ecrg4 plays a critical role in the pathogenesis of AF. However there are limitations in this study: (1) the relatively small sample size in specimens of human atrial appendage may overestimate the observed decrease of Ecrg4 expression in AF, (2) specimens of atrial appendages came from patients with RHD and at least two more other comorbidities, which might have confounded the effect of atrial remodelings caused by loss of Ecrg4, (3) although *in vitro* knockdown ECRG4 seems to suggest that loss of ECRG4 leads to AF, *in vivo* the causal relationship between Ecrg4 and AF remains to be determined, (4) Ecrg4 is a secreted molecule that may affect other cell types than CMs in heart through a paracrine mechanism, which may contribute significantly to atrial remodeling, and (5) whether restoration of Ecrg4 in the canine AF model decreases the incidence of AF and atrial remodeling process remains to be tested. Nevertheless, to our knowledge this is the first report to show that Ecrg4 is involved in the pathogenesis of AF, which may shed new light on a long-held mystery that why the incidence of AF is higher in cancer patients.
